# Supplementary material for: Vero Cells as a Mammalian Cell Substrate for Human Norovirus
Source: Viruses. 2020 Apr 14;12(4):439. doi: 10.3390/v12040439 (PMC7232407; doi:10.3390/v12040439)
Supplement: Supplementary file 1 [file viruses-12-00439-s001.zip › Supplementary Figure 1.pdf]

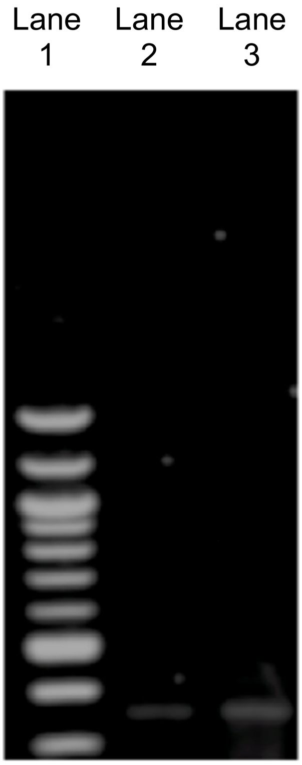

Lane 1: DNA ladder (1500, 1200, 1000, 900, 800, 700, 600, 500, 400, and 300bp markers)
Lane 2: RT-PCR amplified Vero cells + HuNoV 0h time-point RNA
Lane 3: RT-PCR amplified Vero cells + HuNoV 72h time-point RNA

Figure S1. ORF1-2 Junction PCR. Amplification of a 350 bp segment, that spans the ORF1-2 junction, is increased at a 72h infection time-point (lane 3) compared to a 0h input time-point (lane 2). 1% agarose gel stained with ethidium bromide and ran at 80V for 2h.
