# Supplementary material for: Vero Cells as a Mammalian Cell Substrate for Human Norovirus
Source: Viruses. 2020 Apr 14;12(4):439. doi: 10.3390/v12040439 (PMC7232407; doi:10.3390/v12040439)
Supplement: Supplementary file 1 [file viruses-12-00439-s001.zip › Supplementary Figure 3.pdf]

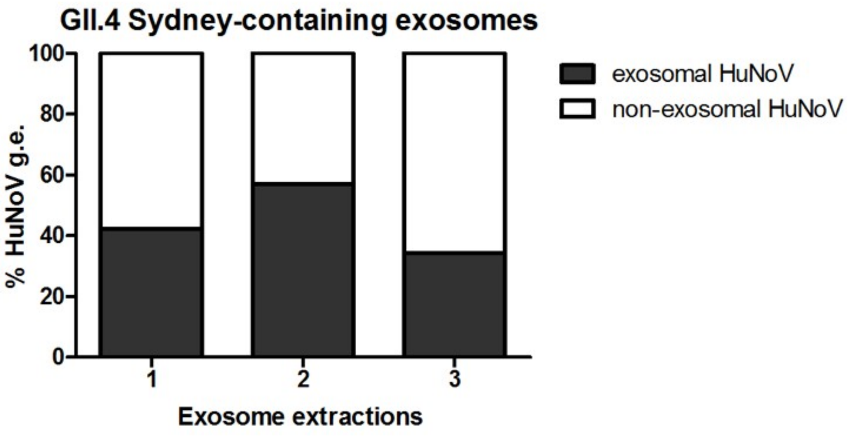

Figure S3. Approximately one-half of HuNoV g.e. in stool are associated with exosomes. Exosomal HuNoV g.e. expressed as a percentage of total expected g.e. content from the stool used for exosome extraction.
