# Supplementary material for: Vero Cells as a Mammalian Cell Substrate for Human Norovirus
Source: Viruses. 2020 Apr 14;12(4):439. doi: 10.3390/v12040439 (PMC7232407; doi:10.3390/v12040439)
Supplement: Supplementary file 1 [file viruses-12-00439-s001.zip › Supplementary Figure 4.pdf]

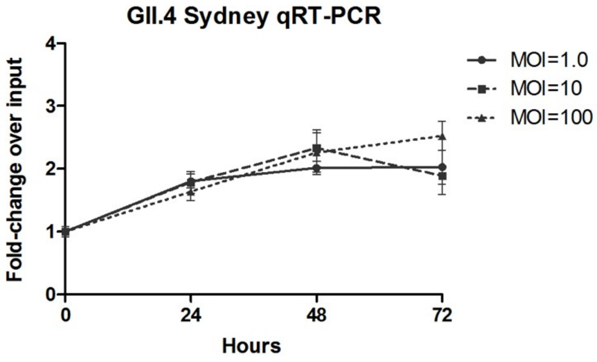

Figure S4. Vero cells support GII.4 Sydney HuNoV across a range of MOIs. The tempo of HuNoV replication is similar regardless of the MOI as quantified by qRT-PCR. Data represent n=3 + SEM.
