# Supplementary material for: Vero Cells as a Mammalian Cell Substrate for Human Norovirus
Source: Viruses. 2020 Apr 14;12(4):439. doi: 10.3390/v12040439 (PMC7232407; doi:10.3390/v12040439)
Supplement: Supplementary file 1 [file viruses-12-00439-s001.zip › Supplementary Figure 5.pdf]

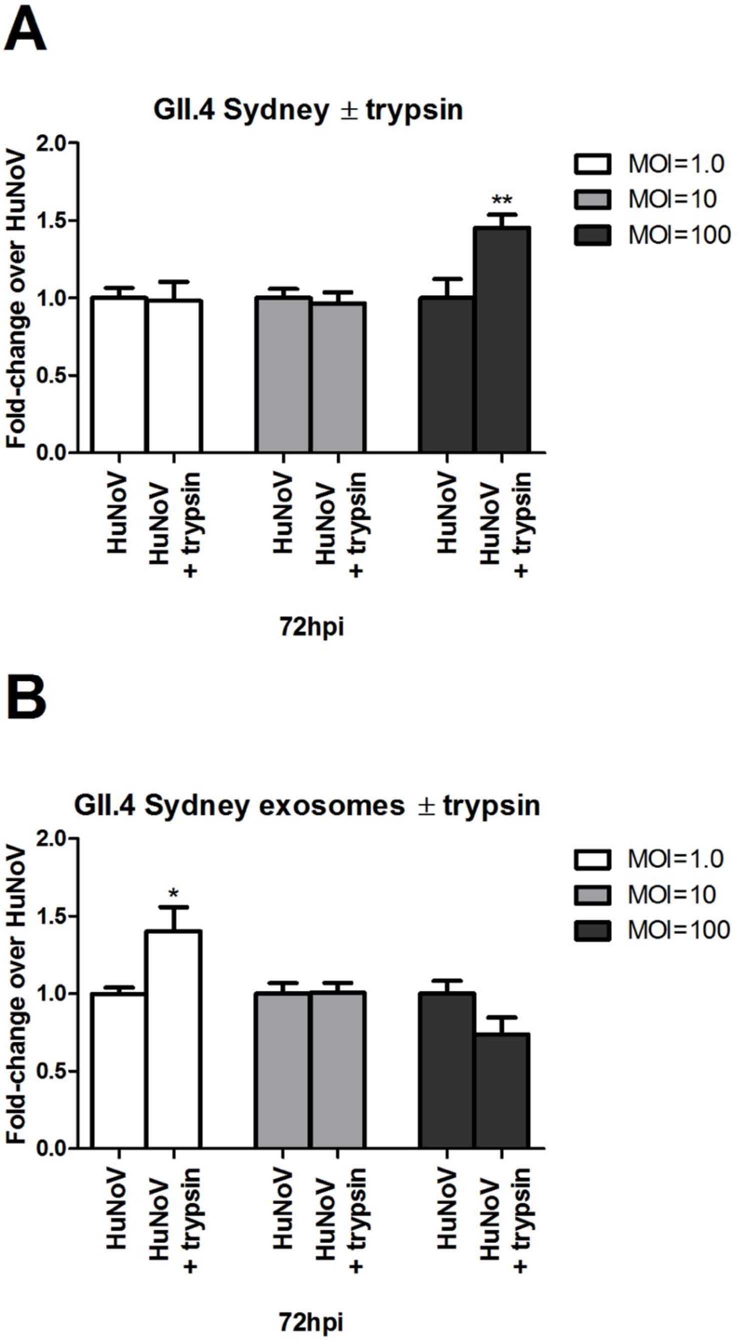

Figure S5. (**A**) Improvement of GII.4 Sydney HuNoV replication by trypsin addition is MOI-dependent. Incubation of 8.8 BAEE units of trypsin with Vero cells increased peak HuNoV levels at a high MOI as measured by qRT-PCR. Data represent n=3 + SEM. ** $p < 0.01$. (**B**) Improvement of exosome-associated GII.4 Sydney HuNoV replication by trypsin addition is MOI-dependent. Incubation of 8.8 BAEE units of trypsin with Vero cells increased peak HuNoV levels at a low MOI as measured by qRT-PCR. Data represent n=2 + SEM. * $p < 0.05$.
