# Supplementary material for: Vero Cells as a Mammalian Cell Substrate for Human Norovirus
Source: Viruses. 2020 Apr 14;12(4):439. doi: 10.3390/v12040439 (PMC7232407; doi:10.3390/v12040439)
Supplement: Supplementary file 1 [file viruses-12-00439-s001.zip › Supplementary Figure 6.pdf]

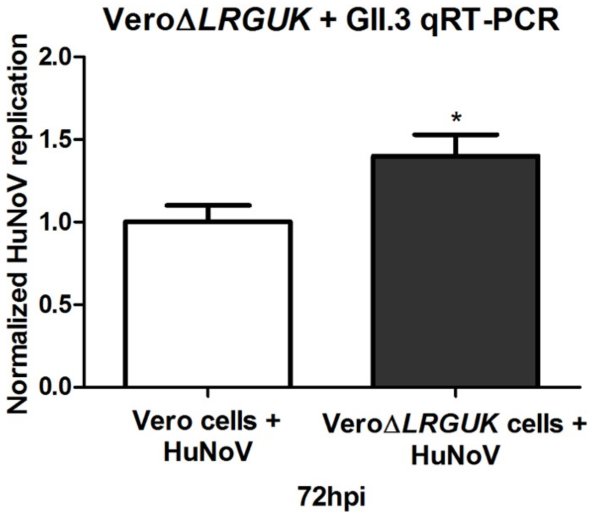

Figure S6. *LRGUK* gene KO in Vero cells improves GII.3 HuNoV replication. Knockout of the *LRGUK* gene from Vero cells improved peak virus loads of GII.3 HuNoV in Vero cells at a 72h time-point. Data represent n=3 + SEM. * $p < 0.05$.
