# Supplementary material for: Vero Cells as a Mammalian Cell Substrate for Human Norovirus
Source: Viruses. 2020 Apr 14;12(4):439. doi: 10.3390/v12040439 (PMC7232407; doi:10.3390/v12040439)
Supplement: Supplementary file 1 [file viruses-12-00439-s001.zip › Supplementary Figure 7.pdf]

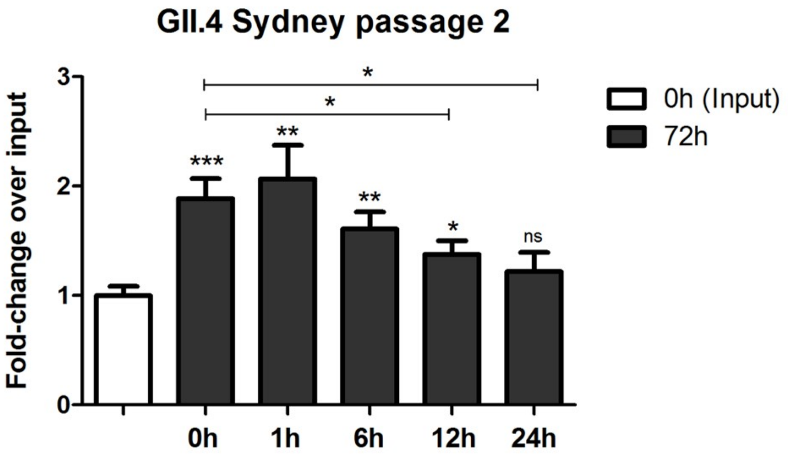

Figure S7. GII.4 Sydney passage is inhibited following prolonged incubation with Vero cells. Incubation of HuNoV with Vero cells for longer than 12h decreases replication potential after passage to fresh Vero cells for 72h. Data represent n=3 + SEM. Not significant (ns), * $p < 0.05$, ** $p < 0.01$, and *** $p < 0.001$.
