# Supplementary material for: Vero Cells as a Mammalian Cell Substrate for Human Norovirus
Source: Viruses. 2020 Apr 14;12(4):439. doi: 10.3390/v12040439 (PMC7232407; doi:10.3390/v12040439)
Supplement: Supplementary file 1 [file viruses-12-00439-s001.zip › Supplementary Table 1.pdf]

Cell lines		Virus strains		Cell Culture Addition		miRs		Vero KO cell lines	
Caco-2 cells	×	GII.3	✓	Bile	–	miR-let7	–	Δ*CNTD2*	–
HEp-2 cells	×	GII.4 Yerseke 2006a	✓	CD300lf plasmid transfection	–	miR-18a	–	Δ*EMX2*	–
HepG2 cells	×	GII.4 Den Haag 2006b	×	Higher pH	–	miR-26b	–	Δ*EP300*	–
INT-407 cells	×	GII.4 Sydney 2012	✓	Higher pH +trypsin	–	miR-105	–	Δ*LRGUK*	✓/–
RAW264.7 cells	×			Lower pH	–	miR-223	–	Δ*NEU2*	–
RAW264.7 + Caco-2 cells	×			Lower pH + trypsin	–	miR-512	–	Δ*WDR62*	–
Vero cells	✓			Synthetic H-antigen	–			Δ*ZNF205*	–
				Trypsin	✓				

Table S1. Summary of HuNoV infection efforts. ✓ indicates successful replication or enhancement of infection. **–** indicates no effect on infection. × indicates no infection. ✓/**–** for VeroΔ*LRGUK* cells indicates that enhanced replication was observed with GII.3 but not GII.4 Sydney or GII.4 Yerseke strains. Abbreviations: cyclin N-terminal domain containing 2 (*CNTD2*), empty spiracles homeobox 2 (*EMX2*), E1A binding protein p300 (*EP300*), leucine-rich repeats and guanylate kinase domain-containing (*LRGUK*), neuraminidase 2 (*NEU2*), WD repeat domain 62 (*WDR62*), and zinc finger protein 205 (*ZNF205*).
