# Supplementary material for: Vero Cells as a Mammalian Cell Substrate for Human Norovirus
Source: Viruses. 2020 Apr 14;12(4):439. doi: 10.3390/v12040439 (PMC7232407; doi:10.3390/v12040439)
Supplement: Supplementary file 1 [file viruses-12-00439-s001.zip › Supplementary Table 2.pdf]

Host gene knockdown							
BTN2A1	–	*IL-10R2*	–	*NAT9*	–	*STRADA*	–
CNTD2	–	*IRF3*	✓	*NDUFA9*	–	*SVOPL*	–
COQ9	–	*IRF7*	✓	*NEU2*	✓	*TLR2*	✓
COX20	–	*IRF9*	–	*PLA2G1B*	–	*TLR3*	✓
EMX2	✓	*JAK1*	–	*PYCR1*	✓	*TLR7*	✓
EP300	–	*JAK2*	–	*RAD51AP1*	✓	*TLR8*	–
FGF2	✓	*LRGUK*	–	*RIG-I*	✓	*TRAF3*	–
GCGR	–	*MAPK6*	–	*SEC61G*	✓	*TYK2*	–
IFNGR1	–	*MAVS*	–	*STAT1*	–	*WDR62*	–
IFNGR2	–	*MDA5*	✓	*STAT2*	–	*ZFYVE9*	–

Table S2. Summary of antiviral host gene knockdown efforts. ✓ indicates the enhancement of infection. **–** indicates no effect on infection. Abbreviations: butyrophilin subfamily 2 member A1 (*BTN2A1*), cyclin N-terminal domain containing 2 (*CNTD2*), coenzyme Q9 (*COQ9*), cytochrome c oxidase assembly factor COX20 (*COX20*), empty spiracles homeobox 2 (*EMX2*), E1A binding protein p300 (*EP300*), fibroblast growth factor 2 (*FGF2*), glucagon receptor (*GCGR*), interferon gamma receptor (*IFNGR*), interleukin 10 receptor 2 (*IL-10R2*), interferon regulatory factor (*IRF*), Janus kinase (*JAK*), leucine-rich repeats and guanylate kinase domain-containing (*LRGUK*), mitogen-activated protein kinase 6 (*MAPK6*), mitochondrial antiviral signaling protein (*MAVS*), melanoma differentiation-associated protein 5 (*MDA5*), N-acetyltransferase 9 (*NAT9*), NADH:ubiquinone oxidoreductase subunit A9 (*NDUFA9*), neuraminidase 2 (*NEU2*), phospholipase A2 group IB (*PLA2G1B*), pyrroline-5-carboxylate reductase 1 (*PYCR1*), RAD51 associated protein 1 (*RAD51AP1*), retinoic acid-inducible gene-I (*RIG-I*), Sec61 translocon gamma subunit (*SEC61G*), signal transducer and activator of transcription (*STAT*), STE20 related adaptor alpha (*STRADA*), SVOP like (*SVOPL*), toll-like receptor (*TLR*), TNF receptor associated factor 3 (*TRAF3*), tyrosine kinase 2 (*TYK2*), WD repeat domain 62 (*WDR62*), and zinc finger FYVE-type containing 9 (*ZFYVE9*).
