# Supplementary figures and images for: Vero Cells as a Mammalian Cell Substrate for Human Norovirus
Source: Viruses. 2020 Apr 14;12(4):439. doi: 10.3390/v12040439 (PMC7232407; doi:10.3390/v12040439)

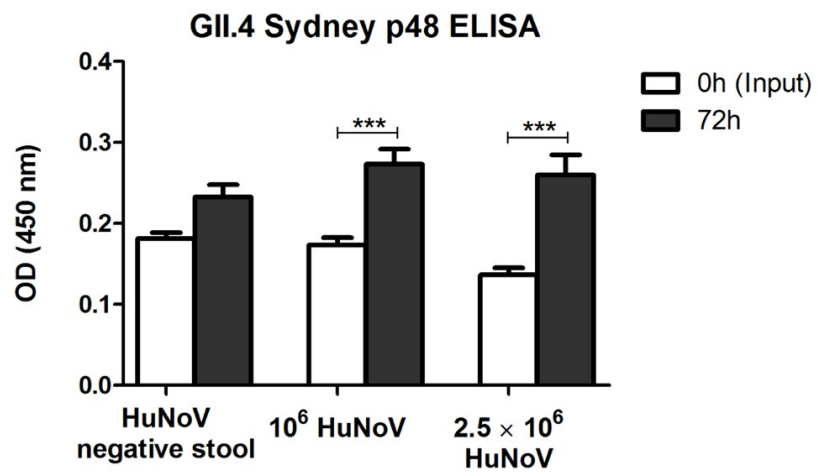

**Figure S2.** Detection of GII.4 Sydney HuNoV p48 by ELISA. Data represent n=3 + SEM.  
\*\*\*  $p < 0.001$

Supplement: Supplementary file 1 [file viruses-12-00439-s001.zip › Supplementary Figure 2.pdf]
